# Supplementary figures and images for: A study on the association between eye movements and regular mouthing movements (RMMs) in normal fetuses between 24 to 39 weeks of gestation
Source: PLoS One. 2020 May 29;15(5):e0233909. doi: 10.1371/journal.pone.0233909 (PMC7259622; doi:10.1371/journal.pone.0233909)

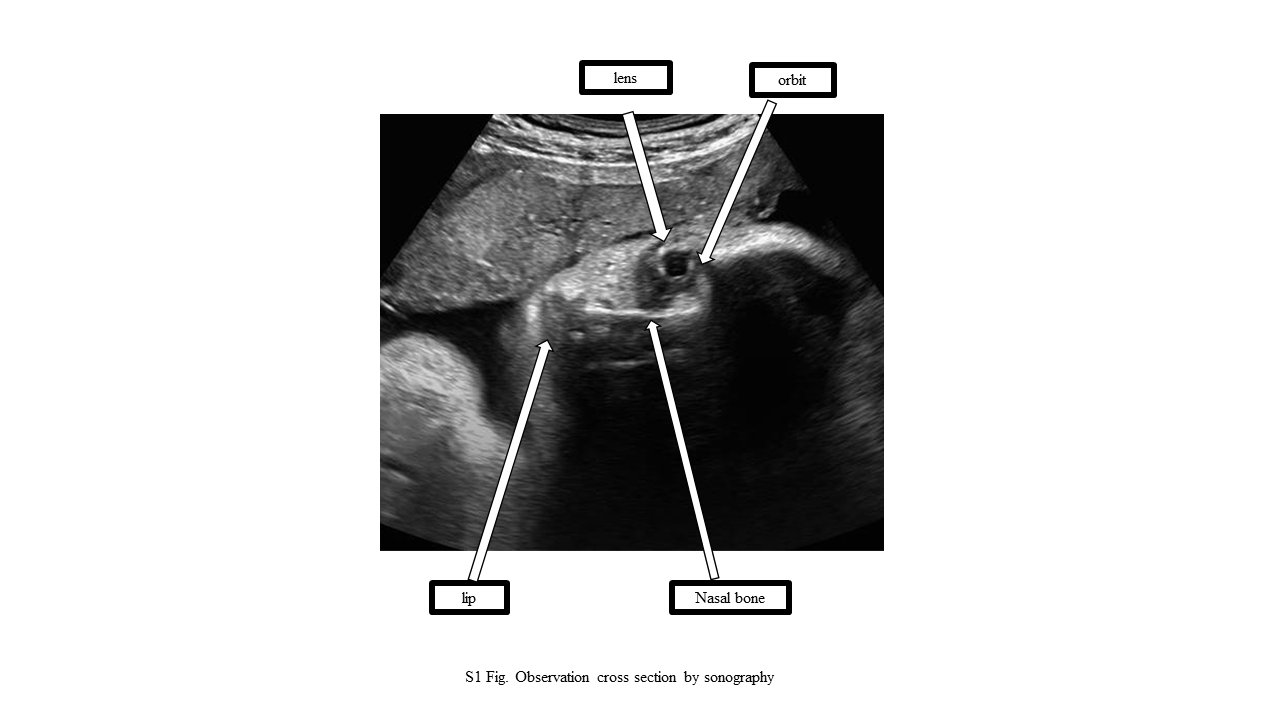

Supplement: S1 Fig — A cross section of the fetal eye movements and mouthing movements observable on coronal imaging in which the edge of the fetal lens was depicted as a ring-shaped circular echoic image at the same time that the mouth was observed. (TIF) [file pone.0233909.s001.tif]

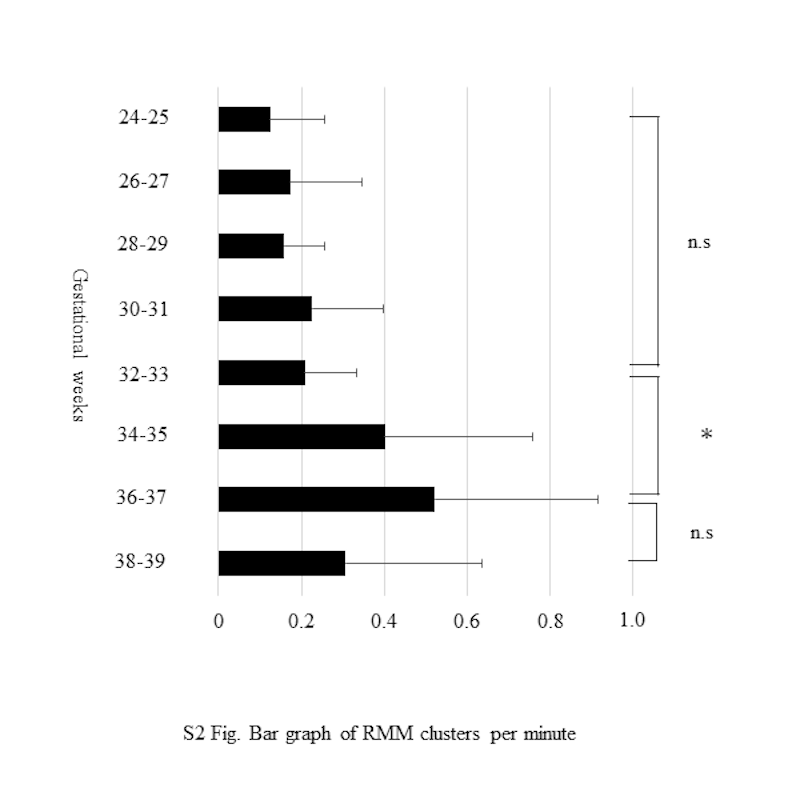

Supplement: S2 Fig — The horizontal axis indicates the RMM clusters per minute and the vertical axis indicates the weeks of gestation. The bars represent means and standard deviations. *p < 0.05, n.s.; not significant. (TIF) [file pone.0233909.s002.tif]

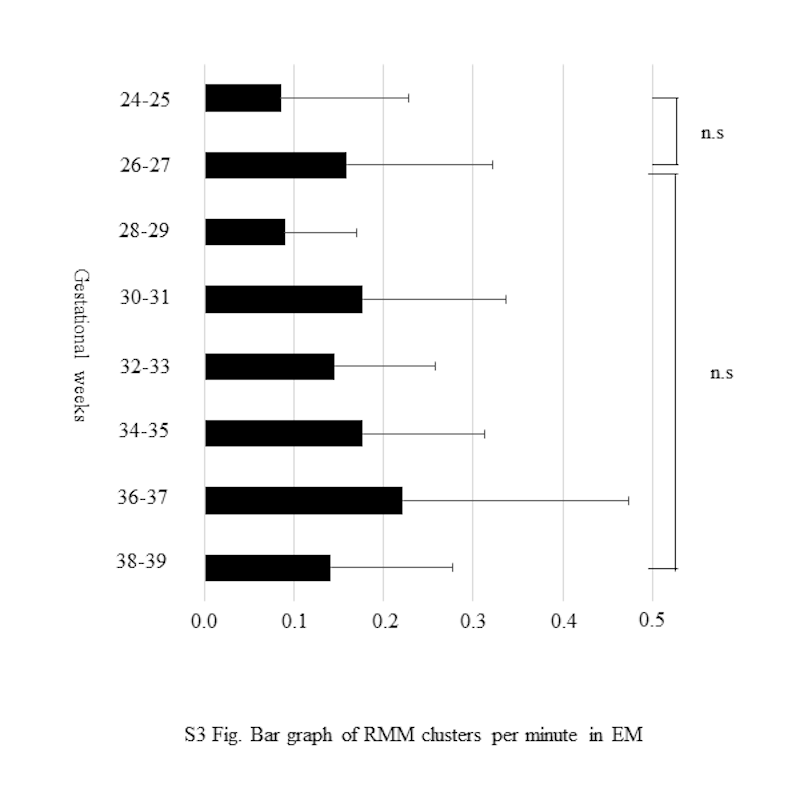

Supplement: S3 Fig — The horizontal axis indicates RMM clusters per minute in EM and the vertical axis indicates gestational weeks. The bars represent means and standard deviations. n.s.; not significant. (TIF) [file pone.0233909.s003.tif]

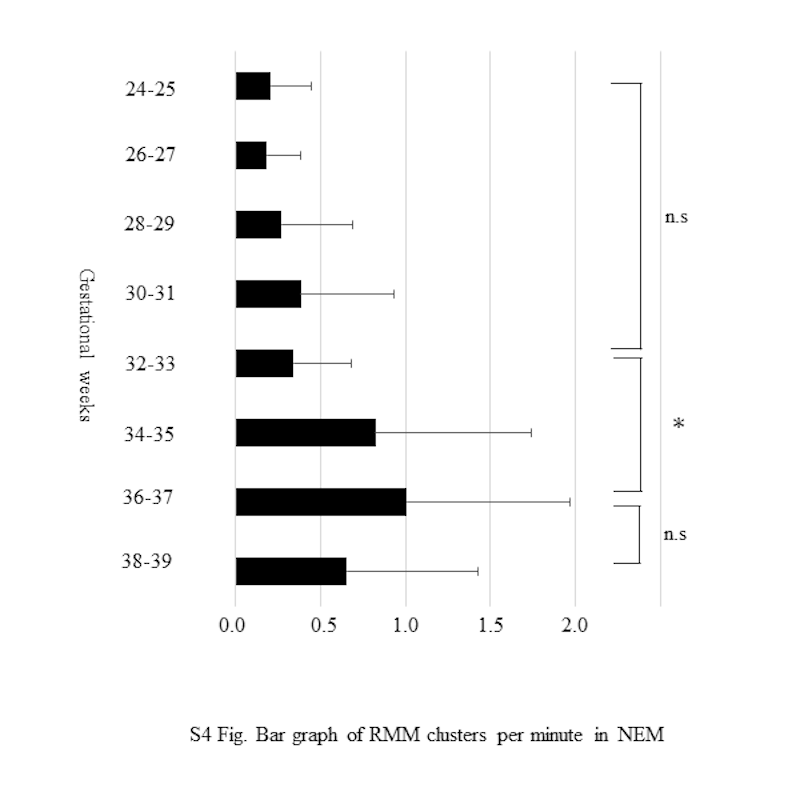

Supplement: S4 Fig — The horizontal axis indicates RMM clusters per minute in NEM and the vertical axis indicates the gestational weeks. The bars represent means and standard deviations. *p < 0.05, n.s.; not significant. (TIF) [file pone.0233909.s004.tif]

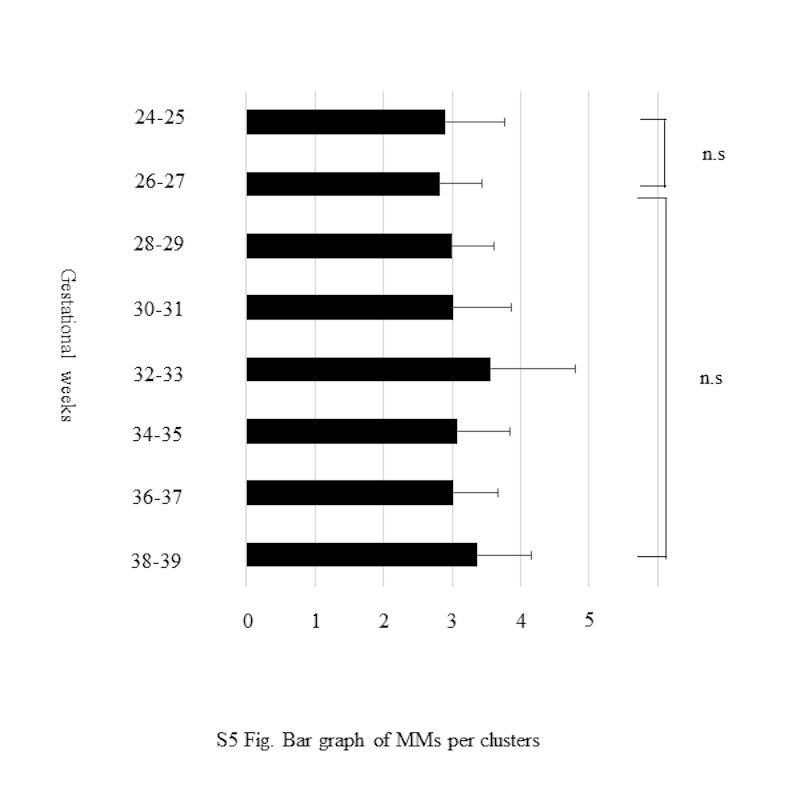

Supplement: S5 Fig — The horizontal axis indicates the MMs per cluster and the vertical axis indicates the gestational weeks. The bars represent means and standard deviations. n.s.; not significant. (TIF) [file pone.0233909.s005.tif]

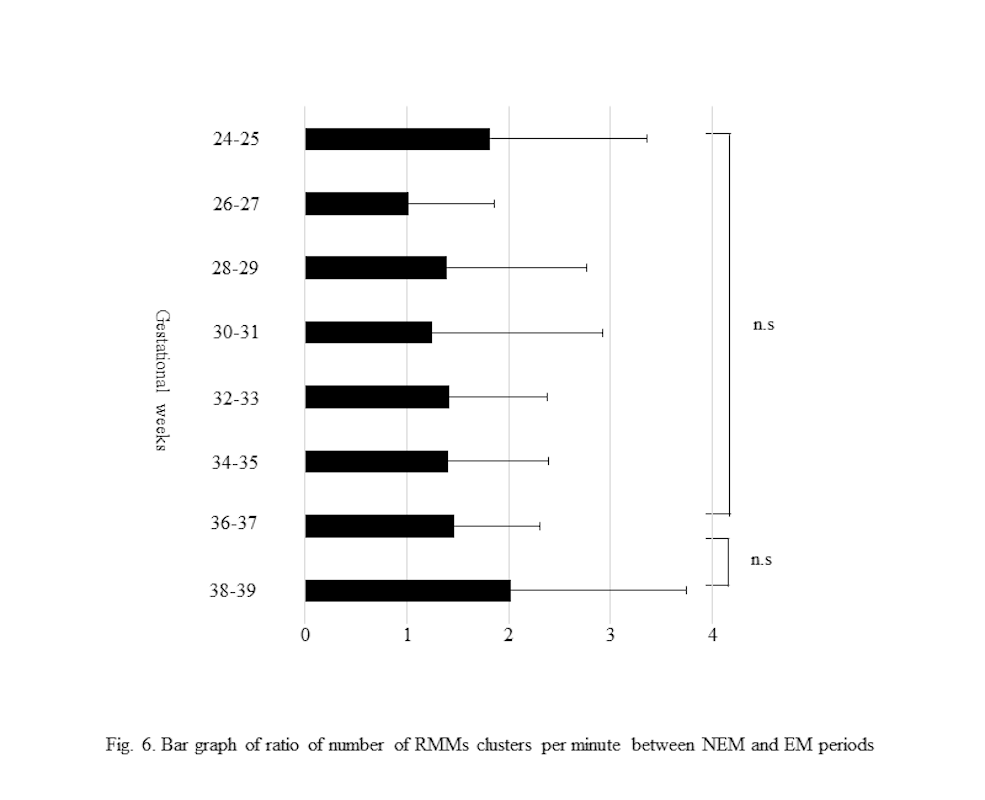

Supplement: S6 Fig — The horizontal axis indicates the ratio of number of RMM clusters per minute between NEM and EM periods and the vertical axis indicates the gestational weeks. The bars represent means and standard deviations. n.s.; not significant. (TIF) [file pone.0233909.s006.tif]
