# Supplementary material for: A study on the association between eye movements and regular mouthing movements (RMMs) in normal fetuses between 24 to 39 weeks of gestation
Source: PLoS One. 2020 May 29;15(5):e0233909. doi: 10.1371/journal.pone.0233909 (PMC7259622; doi:10.1371/journal.pone.0233909)
Supplement: Attachment — Submitted filename: Response_to_Reviewers.docx [file pone.0233909.s007.docx]

**Response to Reviewers**

We would like to thank the Reviewer for taking the time to review our manuscript and for the useful comments. The reviewer comments are in italics below, while our responses are typed in bold. Changes in response to the comments have been highlighted in red font in the revised manuscript.

***Comments to the Author*** *Additional Editor Comments:
My concern about this manuscript is the subjective assessments of mouthing movement and eye movement in this study. Authors should clarify this subject.*

**Response:**

**Thank you for your kind comment. I apologize for the difference between the reference number assignment and the reference list about reference numbers. All references and citations have been re-checked and corrected.**

**In this study, repeat measurements of the tapping process were performed in three cases. The tapping process was performed independently in each case to calculate both inter-observer (M.K. and M.S.) and intra-observer (M.K.) intra-class correlation coefficients (ICCs). The intra- and inter-observer reliability values for the total number of RMM clusters were 0.92–0.95, which indicates a high level of reliability for data recording using the tapping process.**

**The intra- and inter-observer ICC were calculated in reference #16 in the revised manuscript (Okawa H, Morokuma S, Maehara K, Arata A, Ohmura Y, Horinouchi T et al. Eye movement activity in normal human fetuses between 24 and 39 weeks of gestation. PLoS ONE 2017;12(7): e0178722. doi: 10.1371/journal.pone.0178722). We calculated an ICC greater than 0.8, indicating good reliability.**

*Reviewer #1: General comments*

*My big concern is the subjective assessments of mouthing movement and eye movement in this study. How about the inter-observer reproducibility? Authors should address this issue.*

**Response:**

**Thank you for your kind comment. In this study, repeat measurements of the tapping process were performed in three cases. The tapping process was performed independently in each case to calculate both inter-observer (M.K. and M.S.) and intra-observer (M.K.) intra-class correlation coefficients (ICCs). The intra- and inter-observer reliability values for the total number of RMM clusters were 0.92–0.95, which indicates a high level of reliability for data recording using the tapping process.**

**The intra- and inter-observer ICC were calculated in reference #16 in the revised manuscript (Okawa H, Morokuma S, Maehara K, Arata A, Ohmura Y, Horinouchi T et al. Eye movement activity in normal human fetuses between 24 and 39 weeks of gestation. PLoS ONE 2017;12(7): e0178722. doi: 10.1371/journal.pone.0178722). We calculated an ICC greater than 0.8, indicating good reliability.**

*Authors should clearly state the new points in this study. Authors should also explain the cause for difference in fetal mouthing movements between their study and previous studies.*

**Response:**

**To date, no studies have observed the formation of RMM clusters over time. The study by van Woerden et al. [reference #5 in the revised manuscript] was limited to fetuses at 38-40 weeks of gestation. Horimoto et al. [reference #9–––– in the revised manuscript] focused on the interval between mouth movements of the fetus. The authors reported that the number of mouth movements with short intervals increase after 32 gestational weeks, and that the number of mouth movements with short intervals increase during non-eye movement periods after 35 gestational weeks. This study focuses on the characteristics of RMMs forming clusters and their association with non-REM sleep.**

**(lines 66-69)**

*Specific comments
1. How did fetal movements affect the 2D sonographic observations of the fetal face? I think that authors could not observe fetal face during fetal movements.*
**Response:**

**There are times when fetal face cannot be observed due to the movement of the fetus. We added a comment in the text, accordingly.**

**(lines 122-126, 284-288)**

*2. Authors should state that they used two-dimensional sonography.*

**Response:**

**We have revised text as suggested by the reviewer.**

**(Line 103)**

*3. How about the frequency of the probe.*

**Response:**

**The frequency of the probe is observed at 30 Hz. Previous studies have reported that a single eye movement can be as long as 0.06 to 4 seconds, with RMM appearing at intervals of 0.3 to 0.6 seconds.**

*Reviewer #2:*

*There is a minor typo in the abstract (line 24; 'sacking').*

**Response:**

**We have corrected the typographical error in accordance with the comment (‘sucking’).**

**(Line 24)**

*This is a well written manuscript with a clear and concise aim. I do feel that there likely to be a rather loose association between the value of sucking movements as a neurological marker, and that there is too much emphasis placed on the (temporal) association with cortical or synaptic maturation.
There is considerable data emerging on behaviour patterns that involve a much greater integration of the neurological axis, such as the so-called 'General Movements' or behavioural state analysis, which themselves are not the most robust marker of neurological integrity.*
**Response:**

**Thank you for your kind comment. We have revised the text in accordance with the comment.**

**(Lines 275-277)**

*Reviewer #3:*

*1) reading the paper, it seems that there was no movement whatsoever of any fetus leading to failure of recording the eye and mouth movements in the whole series*

**Response:**

**Thank you for your kind comment. There are times when the fetal face cannot be observed due to the movement of the fetus. So I have added explanations as suggested by the reviewer. In the Analytical methods (Data processing) and study limitations sections**

**(lines 122-126, 284-288)**

*2) in advanced gestation it is rather unusual for the fetuses to lie in such a position that the reference view employed in the study - ie coronal view and contemporary observation of lips and eyes (lenses) - is visible. These two factors should be clarified and explained. From my experience, it is virtually impossible that over 60 uninterrupted minutes of observation the fetuses do not show at least some breathing or major trunk (or limb) movements which will hamper or at least interrupt the continuous observation of the fetal face. And the authors have neither reported any failure in the recording nor acknowledged this in the limitations to the study. furthermore, the single paper (published by the same group) cited to account for reproducibilty is cited as ref n.33, but the reference list stops at 31...*

**Response:**

**As you pointed out, continuous observation is sometimes difficult. There are times when fetal face cannot be observed due to the movement of the fetus. Accordingly, I have added this concern to the methods and study limitations.**

**(lines 122-126, 284-288)**

**Further, I apologize for the difference between the reference number assignment and the reference list about reference numbers. All references and citations have been re-checked and corrected.**

**The intra- and inter-observer intra-class correlation coefficients (ICC) were calculated in reference number 16 of the revised manuscript (Okawa H, Morokuma S, Maehara K, Arata A, Ohmura Y, Horinouchi T et al. Eye movement activity in normal human fetuses between 24 and 39 weeks of gestation. PLoS ONE 2017;12(7): e0178722. doi: 10.1371/journal.pone.0178722.) ICC greater than 0.8, indicating good reliability.**

*Finally, all the figures attached should report the titles of the axes ON the image and not only in the caption.*

**Response:**

**I have revised all figures according to the comment.**

*I have doubts about the complete absence or reported failures in the recordings.
To improve the manuscript, this major flaw should be addressed in the discussion and in the presentation of data, and a figure /clip illustrating the sonographic approach should be included*

**Response:**

**I have added that there were times when observation was impossible due to the movement of the fetus, and how to use the data at that time.**

**(lines 122-126)**

**Furthermore, I have added the limitations of the method in the limitations section.**

**(lines 284-288)**

**Finally, I have added to a figure/clip illustrating the sonographic approach as S1 Fig. Accordingly, S1-S5 Figs have been changed to S2-S6 Fig. And I have added to sporting information about S1 Fig.**

**(lines 386-389)**

*Reviewer #4:*

*can the authors make a comment on the pregnant women characteristics including maternal data; presence of preeclampsia, maternal disease, chorioamnionitis and etc.*

*Were the mothers have these kind of problems, or all were healthy pregnant women?*

**Response:**

**Thank you for your kind comment. I have revised the text from “Pregnancies with fetal morphological abnormalities or maternal complications were excluded.” to “Cases with apparent fetal morphological abnormalities and maternal complications at the time of recruitment were excluded. However, after data collection, as the pregnancy progressed, there were 5 cases diagnosed with gestational diabetes mellitus and 5 cases diagnosed with hypertensive disorders of pregnancy.”**

**(lines 86-89)**

*Can they comment on the clinical practical use of these data on both antenatal and postnatal evaluation in both fetuses and infants, respectively?*

**Response:**

**By continuously observing the infant after birth, it may be possible to confirm the developmental prognosis of the baby in relation to RMM. Therefore, there is a possibility that these data can be clinically used as one of the indicators of neurodevelopment in the future.**

**We added this content in the revised manuscript.**

**(lines 278-281)**
