# Supplementary material for: A study on the association between eye movements and regular mouthing movements (RMMs) in normal fetuses between 24 to 39 weeks of gestation
Source: PLoS One. 2020 May 29;15(5):e0233909. doi: 10.1371/journal.pone.0233909 (PMC7259622; doi:10.1371/journal.pone.0233909)
Supplement: Attachment — Submitted filename: PONE-D-19-33770R1-(3-27-20).doc [file pone.0233909.s008.doc]

***To the Editor***

Thank you for giving me the opportunity of reviewing the revised manuscript submitted to the **PROS ONE**. The revised manuscript is significantly improved. However, I think that one minor revision is needed to accept this manuscript in the **PROS ONE**.

# *Comment to the authors*

**General comments**

The revised manuscript is significantly improved. However, I think that one minor revision is needed to accept this manuscript in the **PROS ONE**.

**Specific comments**

1. The frequency of the probe means 3-MHz, 3,5-MHz, or 5-MHz, etc. Authors should state the frequency of the probe in the text.
