# Supplementary material for: A study on the association between eye movements and regular mouthing movements (RMMs) in normal fetuses between 24 to 39 weeks of gestation
Source: PLoS One. 2020 May 29;15(5):e0233909. doi: 10.1371/journal.pone.0233909 (PMC7259622; doi:10.1371/journal.pone.0233909)
Supplement: Attachment — Submitted filename: Responce_to_Reviewers_20200426.docx [file pone.0233909.s009.docx]

**Response to Reviewers**

We would like to thank the reviewers for taking the time to review our manuscript and for their useful comments. The reviewers' comments are in italics below, while our responses are typed in bold. Changes in response to the comments have been highlighted in red font in the revised manuscript.

6. *Review Comments to the Author

Please use the space provided to explain your answers to the questions above. You may also include additional comments for the author, including concerns about dual publication, research ethics, or publication ethics. (Please upload your review as an attachment if it exceeds 20,000 characters)

Reviewer #1: General comments

The revised manuscript is significantly improved. However, I think that one minor revision is needed to accept this manuscript in the PROS ONE.

Specific comments

1. The frequency of the probe means 3-MHz, 3,5-MHz, or 5-MHz, etc. Authors should state the frequency of the probe in the text.*

**Response: Thank you for your kind comments. The frequency of the probe used is 3.5MHz. We have added the frequency of the probe in the text. (Line 100-101)**

*Reviewer #2: The authors have made a good effort to address the comments from all the reviewers, and I am happy to support the publication*

**Response: Thank you for this kind comment; we appreciate your support.**

*Reviewer #3: The authors have submitted a new version of the manuscript, apparently revised according to the comments of the reviewers which they claim were "fully considered and included in the revised version". However, at least for the points I raised, they only reported a very generic sentence "Cases where such observation was not possible or time during which observation was not possible were not included". The authors did neither mention the number of cases in which the recording had started and then aborted nor their incidence by weeks of pregnancy. In addition, they did not include a sonographic image to show the technique, nor a clip to this extent.
Since the former was my major criticism, because it is quite clear tha major head and trunk movements do occur quite frequently in the 3rd trimester of pregnancy, I do not think my comments were adequately dealt for. Therefore, I would recommend rejection of the paper, due to possible selection bias and failure to comply with reviewers' suggestions*

**Response: Thank you for your comment.** **We apologize for not fully answering your concerns in the last round of revision. In two cases, the effective observation time was insufficient due to fetal posture and movement; the gestational weeks were 36 and 38 weeks, respectively, when the two cases were observed. We apologize for not including these two examples in the fetal population. Therefore, we have revised the manuscript according to your comment (line 176-178). When we were unable to render the appropriate cross-section, we adjusted the position of the probe and asked the mother to change her position so that the proper cross-section could be rendered. We have revised the manuscript to clarify this (line 105-107). We have also described this as a limitation of the study (line 286-287).**

**In addition, we have added a figure /clip illustrating the sonographic approach as S1 Fig. Finally, many other researchers have used this research method. Reference number 5, 9, and 16 use a similar research method to the one used in our study.**

*Reviewer #4: Thank you for the revisions
The authors made all the changes that have been recommended by the reviewers*

**Response: Thank you for this kind comment; we appreciate your support.**
